# *Allium* Extract Implements Weaned Piglet’s Productive Parameters by Modulating Distal Gut Microbiota

**DOI:** 10.3390/antibiotics10030269

**Published:** 2021-03-08

**Authors:** Miguel Rabelo-Ruiz, Claudia Teso-Pérez, Juan Manuel Peralta-Sánchez, Juan José Ariza, Antonio Manuel Martín-Platero, Óscar Casabuena-Rincón, Patricia Vázquez-Chas, Enrique Guillamón, María Arántzazu Aguinaga-Casañas, Mercedes Maqueda, Eva Valdivia, Alberto Baños, Manuel Martínez-Bueno

**Affiliations:** 1 Departamento de Microbiología, Universidad de Granada, Avda. Fuentenueva, s/n, 18071 Granada, Spain; claudiatp@ugr.es (C.T.-P.); jmps@ugr.es (J.M.P.-S.); ammartin@ugr.es (A.M.M.-P.); mmaqueda@ugr.es (M.M.); evavm@ugr.es (E.V.); mmartine@ugr.es (M.M.-B.); 2 Departamento de Microbiología y Biotecnología, DMC Research Center, Camino de Jayena s/n, 18620 Granada, Spain; jariza@dmcrc.com (J.J.A.); eguillamon@domca.com (E.G.); arancha.aguinaga@domca.com (M.A.A.-C.); abarjona@dmcrc.com (A.B.); 3 IMASDE AGROALIMENTARIA S.L., 28224 Pozuelo de Alarcón, Madrid, Spain; ocasabuena@e-imasde.com (Ó.C.-R.); pvazquez@e-imasde.com (P.V.-C.); 4 Instituto de Biotecnología, Universidad de Granada, Avda. Fuentenueva, s/n, 18071 Granada, Spain

**Keywords:** *Allium* extract, bacterial community, high-throughput sequencing, phytobiotic, piglet microbiome, productive parameters

## Abstract

Antimicrobial resistance (AMR) has risen as a global threat for human health. One of the leading factors for this emergence has been the massive use of antibiotics growth-promoter (AGPs) in livestock, enhancing the spread of AMR among human pathogenic bacteria. Thus, several alternatives such as probiotics, prebiotics, or phytobiotics have been proposed for using in animal feeding to maintain or improve productive levels while diminishing the negative effects of AGPs. Reducing the use of antibiotics is a key aspect in the pig rearing for production reasons, as well as for the production of high-quality pork, acceptable to consumers. Here we analyze the potential use of *Allium* extract as an alternative. In this study, weaned piglets were fed with *Allium* extract supplementation and compared with control and antibiotic (colistin and zinc oxide) treated piglets. The effects of *Allium* extract were tested by analyzing the gut microbiome and measuring different productive parameters. Alpha diversity indices decreased significantly in *Allium* extract group in caecum and colon. Regarding beta diversity, significant differences between treatments appeared only in caecum and colon. *Allium* extract and antibiotic piglets showed better values of body weight (BW), average daily weight gain (ADG), and feed conversion ratio (FCR) than control group. These results indicate that productive parameters can be implemented by modifying the gut microbiota through phytobiotics such as *Allium* extract, which will drive to drop the use of antibiotics in piglet diet.

## 1. Introduction

Antibiotics have been used to promote growth and production in livestock (Antibiotic Growth Promoters, AGP [1,2]). However, the inappropriate and indiscriminate use of them contributed to a rising of resistance to antibiotics [3]. This situation drove the World Health Organization (WHO) to call for a global action against Antimicrobial Resistance (AMR; [4]). For this reason, AGPs are banned by the European Union since 2006 (EC Regulation 1831/2003; http://eur-lex.europa.eu/en/index.htm) and by other countries during following years [5,6]. However, this ban has produced an increase in mortality, especially at weaning when many stressors affects piglets’ health, leading to an increase of post-weaning diarrhea caused by *Escherichia coli* infections [7,8]. This increase in mortality directly affects the pork industry, as pork and its derivatives are product highly consumed daily throughout the world [9]. In this sense, reducing the use of antibiotics is a key aspect in the pig rearing for production reasons, but also for the production of high-quality pork, acceptable to consumers.

The mechanisms through which AGPs act are not very clear but it is believed that growth promotion could be associated with changes in the gut microbiota [10,11]. AGPs may favor the reduction of pathogenic bacteria, the reduction of bacterial competition for nutrients, and reduction of microbial compounds, which can decrease animal growth [12,13]. However, the use of AGPs has undesirable effects such as selection and spread of antibiotic resistance genes [14]. Some studies show evidence of the occurrence of AMR in relation to the use of antibiotics in cattle and specifically in the swine industry [15,16]. Many bacterial strains resistant to a wide variety of antibiotics have been found in the intestinal microbiota of pigs, such as *Campylobacter coli, C. jejuni, Salmonella,* or the multiresistant *Staphylococcus aureus* (livestock-associated MRSA) [14,17,18,19]. Given this problem of AMR and the subsequent ban of AGPs in food animal production, there has been a need to look for alternatives that maintain animal health and increase productive levels of pigs while decreasing the use of antibiotics [2,20].

Different compounds have been proposed as substitutes to AGPs in swine industry improving health and performance of pigs. Probiotics, prebiotics, organic acids, enzymes, or phytobiotics have been widely recognized as promising alternatives to antibiotics in feeds [2]. Phytobiotics are plant-derived products used in animal feed to improve performance of livestock. Some studies had demonstrated their antimicrobial, antioxidants, and immunoregulatory effects in poultry and pigs [2,21]. Given these positive properties of phytobiotics, several researchers have tried to demonstrate that their inclusion in diets can improve pig performance. Some studies have shown positive results using different plant extracts including oregano oil [22,23], menthol and cinnamon [22,24], a mixture of different plant extracts [25,26], and garlic [27]. Garlic had also been used due to its antifungal, antimicrobial, and antioxidant properties [28,29]. Currently, several active organosulfur compounds extracted from garlic and other *Allium* plants, such as PTS (propyl propane thiosulfinate) and PTSO (propyl propane thiosulfonate), have been characterized [30]. An *Allium* extract, which includes these compounds, has shown high antimicrobial activity against *Salmonella*, *E. coli*, *Clostridium*, methicillin-resistant Staphylococcus aureus (MRSA), *Campylobacter jejuni,* and *Aspergillus* pathogens [31,32]. This product had been mainly used in broiler chickens, modulating intestinal microbiota, improving nutrient digestibility, and reducing pathogens and potentially pathogenic bacteria in the intestinal content [31,33]. PTS and PTSO had also been add to pig diet and showed antimicrobial activity against different bacterial groups, decreasing fecal counts of *Enterobacteriaceae* and coliforms [30].

The aim of the present study was to evaluate the influence of the *Allium* extract in weaned piglet gut microbiota and how it affects productive parameters such as body weight, daily weight gain, daily feed intake, and feed conversion rate. In this study, we have made a fully randomized experiment using piglets as research animal model supplemented with the *Allium* extract. We have characterized the microbiota in different gut regions by high-throughput sequencing of 16S rRNA gene at 70 days of life. We suggest that this phytobiotic compound improves piglet productive parameters by means of distal gut microbiota modification.

## 2. Materials and Methods

### 2.1. Piglets and Farm Facilities

The experiment was carried out at IMASDE AGROALIMENTARIA S.L. in Granja La Mata (Experimental Authorization Ref No: B-82334855), a swine experimental farm situated in Mata de Cuellar (Segovia, Spain). A total of 240 piglets (50% female, 50% male) were used in the experiment. Piglets were housed in a non-litter housing system consisting of 2 rooms, using a total of 24 blocks (12 of each room). Ten crossbred piglets of the same sex (50% Pietrain × 25% Landrace* 25% Large White) from commercial genetic breeds were kept per block of 6.05 m^2^ (2.16 × 2.80 m^2^). Piglets were from stress-free parents. The rooms had natural and artificial lighting, and the temperature was adjusted according to the piglet age. Piglets were weaned at 28 days of life, with an average weight of 7.34 ± 0.89 kg. The farm fulfilled the national regulations and the European directive for the protection of animal welfare in research (Directive 2010/63/EU, European Commission, 2010).

### 2.2. Experimental Design and Sample Collection

Before starting the experiment, animals were examined and those with signs of illness or injury were removed. Subsequently, groups of 10 piglets of the same sex were assigned randomly to different blocks (8 blocks per treatment, 4 in each room). Piglets were regularly monitored during rearing. No signs of loss of weight, abnormal behaviors or deaths were detected. Control piglets were fed with a basal diet, while experimental piglets received basal diet supplemented with *Allium* extract (equivalent to 20 mg/kg of thiosulfinates and thiosulfonates). This *Allium* extract is commercialized under the trademark of Garlicon (DOMCA S.A.U., Spain), and the applied dose is the recommended by the product leaflet. In addition, another group received basal diet supplemented with 120 mg/kg of the antibiotic colistin (Nipoxyme 100) and 3000 ppm of zinc oxide (ZnO) as positive control (colistin was only used for research purpose because it is prohibited for commercial purpose). Basal diet differed in pre-starter (28 to 42 days) and starter (43 to 70 days) (Supplementary Material: Table S1). Both diets and water were supplied ad libitum. Diets were formulated by IMASDE AGROALIMENTARIA S.L. and produced at the factory Gireporc S.A. in Bernuy de Porreros (Segovia, Spain).

Piglets were weighted at weaning (beginning of the experiment—28 days old), at 42 and 70 days old. Other productive parameters were recorded at the end of each experimental stage (42 and 70 days old): Average daily feed intake, ADFI; Average daily weight gain, ADG; and Feed conversion rate, FCR (ADFI divided by ADG). At the end of the experiment (70 days old), one piglet per block (a total of eight piglets of each treatment) was slaughtered by previous electrically stunned and bleed, according to the standardized procedures of slaughterhouse "El cochinillo segoviano" S.L. (Boceguillas, Segovia, Spain). Immediately, pieces of about 10 cm were dissected from different intestinal regions (duodenum and ileum from small intestine; caecum and colon from large intestine) with sterile material. Intestinal pieces were stored in sterile containers and transported to the laboratory, where they were kept at -80 ºC until DNA extraction. Intestinal pieces from different gut regions of piglets were dissected using a sterile scalpel and approximately 100 mg of gut content were collected.

### 2.3. DNA Extraction

DNA extraction was carried out using FavorPrep Stool DNA Isolation Mini Kit (Favorgen Biotech Corp., Taiwan), according to manufacturer instructions. DNA extraction was checked by 0.7% agarose gel electrophoresis and DNA concentration was measured using NanoDrop 2000 Spectrophotometer (Thermo Fisher Scientific, USA). Samples were standardized at the same DNA concentration (10 ng/µL) and then stored at −20 °C until DNA amplification.

### 2.4. High-Throughput Sequencing

Amplicon PCR was performed from bacterial total DNA of the V4 region of the 16S rRNA gene using the primer pair U515F (5´ TCGTCGGCAGCGTCAGATGTGTATAAGAGACAGGTGCCAGCMGCCGCGGTAA-3´) and E786R (5´-GTCTCGTGGGCTCGGAGATGTGTATAAGAGACAGGGACTACHVGGGTWTCTAAT-3´) with overlap partial Illumina primers. This PCR was carried out in a final volume of 25 µL containing 12.5 µL of iProof High-Fidelity DNA Polymerase (Bio-Rad Laboratories, Inc.), 0.3 µM of each primer, and 5 µL of template DNA. The amplification program consisted of an initial denaturing step of 98 °C for 1 min followed by an amplification step of 25 cycles of 10 s at 98 °C, 20 s at 52 °C, and 15 s at 72 °C, and a final extension of 5 min at 72 °C. Then, a second PCR was applied to include specific barcodes by adding a unique combination of a couple of barcodes per sample. This PCR was carried out in a final volume of 25 µL containing 12.5 µL of iProof High-Fidelity DNA Polymerase (Bio-Rad Laboratories, Inc.), 0.4 µM of each primer, and 5 µL of purified PCR product from the previous PCR. The amplification program consisted of an initial denaturing step of 98 °C for 1 min followed by an amplification step of 8 cycles of 10 s at 98 °C, 20 s at 55 °C, and 15 s at 72 °C, and a final extension of 5 min at 72 °C. Purification steps were made using magnetic microparticles with a surface functional group to which DNA can be reversibly linked. Subsequently, the DNA of the magnetic particles were separated by elution [34]. Then, DNA concentration was measured using Qubit^®^ 3.0 Fluorometer (Invitrogen, Carlsbad, CA, USA) and normalized to the same concentration. High-throughput sequencing was carried out on Illumina MiSeq platform in the Scientific Instrumental Center at the University of Granada (CIC-UGR, Spain). Sequences are available in the Genbank-NCBI Sequence Read Archive (SRA) (https://www.ncbi.nlm.nih.gov/sra/), BioProject: PRJNA664026, Accession Nos. SAMN16192455 to SAMN16192544. 

### 2.5. Sequences Processing and Data Analysis

The processing of the sequences obtained from Illumina MiSeq was carried out with QIIME2 v2018.02 (Quantitative Insights In Microbial Ecology [35,36]). First, primers trimming were performed using default parameters using cutadapt plugin [37]. Forward reads were selected for the following analysis due to low quality in reverse reads after 120 bp (Phred score < 20). Quality filtering were performed using default parameters. Afterwards, we used Deblur for sequence clustering into sub-OTUs, a sub-operational-taxonimic-unit (sOTU) approach, in order to remove sequencing errors [38]. Sequences that passed quality filters were truncated to 200 bp, using Phred score of 20 as quality threshold, giving a dataset of 6,548,564 total reads with a mean depth of 70,415 reads per sample. We used fragment insertion script adapted to QIIME2 through the SATé-enabled phylogenetic placement (SEPP) technique, a script that performs the alignment of the sequences and the phylogenetic tree [39]. Taxonomy assignation was made with a classifier pretrained on Greengenes 13.08 with a similarity of 99% [40]. Finally, because the primers used are designed for bacteria, chloroplasts, mitochondria, and non-bacterial DNA were removed from the sOTU table.

### 2.6. Statistics

To test the effect of treatment on production parameters of pigs, we used Generalized Linear Mixed-Models (GLMM). We used 24 experimental units (2 rooms of 12 experimental units each) with treatment as fixed factor, sex, and room as random factors, and initial body weight as covariate.

For alpha and beta diversity analyses, sOTU table was rarified at 17,000 sequences depth per sample. Samples that did not reach this sequencing depth were excluded for subsequent analyses. Two alpha diversity indices were calculated, i.e., bacterial species richness, as number of observed species; and Faith’s phylogenetic diversity index [41]. We used General Linear Models (GLM) to explore the effect of treatment and gut region in different alpha diversity indices. Piglet was used as experimental unit for alpha and beta diversity analysis.

Productive parameters and alpha diversity analyses were performed in Statistica 10.0 (StatSoft).

Beta diversity distance matrixes were calculated using UniFrac distance [42]. In subsequent analysis, we used both Weighted UniFrac and Unweighted UniFrac distance matrixes as we do not have a priori predictions in the effects of the independent variables (gut region and treatment) in the bacterial community. Weighted UniFrac gives more importance to most abundant bacteria as it takes into account the abundance of sequences per sOTU, while Unweighted UniFrac gives the same importance to all bacterial sOTU presents in the samples, giving more importance to minority bacteria as it takes into account the presence or absence of sOTU [43]. Permutational ANOVA (PERMANOVA) based on Type III sums of squares with 999 permutations was used to test treatment and gut region effects on both UniFrac distance matrixes [44] using PRIMER-7 (PRIMER-e). Principal Coordinates Analysis were calculated and visualizations of the first three axes of the PCoA were plotted using Emperor 2018.2.0 [45].

## 3. Results

### 3.1. Effects of Teatment on Piglets’ Gut Bacterial Alpha Diversity

**Figure 1:**
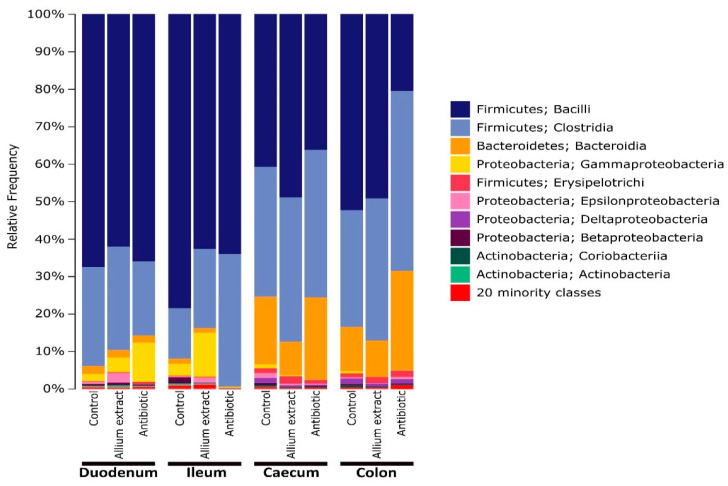
Microbial composition at class level of piglet gut microbiota grouped by gut region and treatment. Classes in the legend are sorted from most abundant to lowest abundant.

Duodenum and ileum microbiota of 70 days control piglets were mainly dominated at classes Bacilli and Clostridia, representing more than 90% between both groups. This pattern was similar in *Allium* extract and antibiotic groups, but with a lower proportion of Bacilli and higher proportion of Gammaproteobacteria in duodenum (10.5% and 3.9% in antibiotic and *Allium* extract group respect to 1.9% in control group) and *Clostridia* in ileum (35.3% and 21.1% in antibiotic and *Allium* extract group respect to 13.5% in control group) (Figure 1). At genus level, duodenum and ileum community of piglets was very diverse, dominated by *Lactobacillus* (more than 65%), followed by an unidentified genus of the family *Clostridiaceae* (6.7%), *Sarcina* (5.9%), *Streptococcus* (3%), and an unidentified genus of the family *Peptostreptococcaceae* (2.8%). Duodenum microbiota was very similar in three groups, but in the ileum, more differences appeared, with lower proportion of Lactobacillus in both *Allium* extract and antibiotic group (Supplementary material: Figure S1). However, no statistically significant differences appeared between treatments in duodenum and ileum in neither Species richness (LSD Posthoc test, *p* > 0.314) nor Faith’s diversity index (LSD Posthoc test, *p* > 0.253).

Large intestine (caecum and colon) microbiota showed a shift in dominant classes respect to small intestine, with lower proportion of Bacilli and higher proportion of Clostridia and Bacteroidia (Figure 1). Caecum microbiome had a very similar distribution in piglets from different treatments, with a slightly higher proportion in *Allium* extract fed piglets of Bacilli (48.9% respect to 40.8% in control group) and lower proportion of Bacteroidia (9.0% respect to 18.1% in control group). At the class level, colon microbiome of control and *Allium* extract groups were very similar, but the Antibiotic group microbiome showed a lower proportion of Bacilli (20.5% compared to 52.3% in control group) and higher proportion of Clostridia and Bacteroidia (Figure 1). At genus level, caecum microbiome of piglets from different treatments was similar, but in colon region, differences appeared in the antibiotic group, with lower proportion of *Lactobacillus* (14.2%) with respect to control and *Allium* extract groups (48.9 and 46.5%, respectively) and higher proportion of *Prevotella* and the rest of minority genera (Additional file 1: Figure S1). Regarding alpha diversity indices, in caecum, *Allium* extract group showed lower values of Species richness and Faith’s diversity index than Control group (LSD Posthoc test, *p* = 0.007; LSD Posthoc test, *p* = 0.034 respectively). In colon, *Allium* extract group had lower values of Species richness and Faith’s diversity index than antibiotic group (LSD Posthoc test, *p* = 0.008; LSD Posthoc test, *p* = 0.019 respectively).

**Figure 2:**
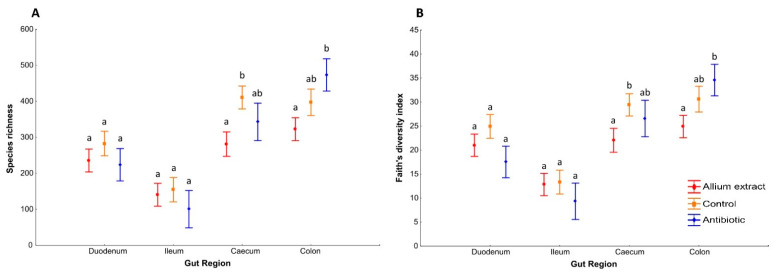
Alpha diversity by gut region. Average ± standard error of the mean of the bacterial species richness (**A**) and Faith’s diversity index (**B**) of weaned piglets in different gut regions. Bars with different letter within the same gut region denote significant differences in treatment (LSD Posthoc test; *p* < 0.05).

Therefore, none of the small intestine region (duodenum and ileum) showed differences between treatments in Species richness and Faith diversity indices, but significant differences in these alpha diversity indices appeared in large intestine regions (caecum and colon) (Figure 2). Taking into account the whole gut, species richness and Faith’s diversity index differed significantly between treatments and between gut regions (Table 1). However, interactions between treatments and gut region were not significant, indicating that alpha diversity indices along the piglets’ gut of different treatments changed in the same way (see interaction Gut Region and Treatment in Table 1).

### 3.2. Effects of Treatment and Gut Region on Beta Diversity

**Figure 3:**
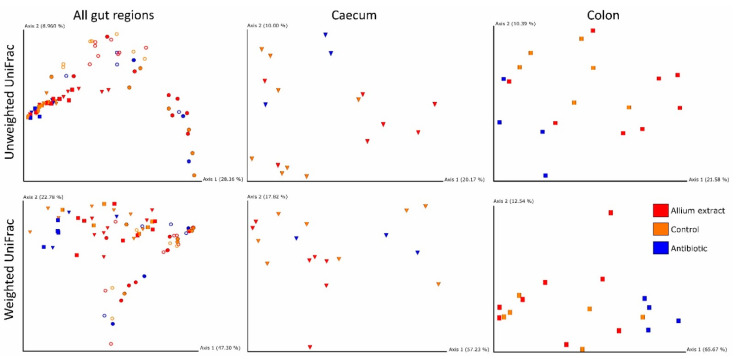
Dimensional figures showing the first two axes of Principal Coordinate Analysis and representing bacterial communities of weaned piglets in all gut regions and taking into account only caecum and colon using Unweighted and Weighted UniFrac distance matrixes. Samples are colored by treatment (Control—yellow; Antibiotic—blue; *Allium* extract —red) and samples from each intestinal region are represented by different shapes (Duodenum—ring; Ileum—sphere; Caecum—cone; Colon—square). Proportion of explained variance by each PCo axes is shown.

Changes in bacterial communities along different piglets’ gut regions were similar in the three experimental groups (see non-significant interaction terms Gut Region*Treatment of both Unweighted and Weighted UniFrac in Table 2). However, Gut Region and Treatment had a significant effect on the intestinal microbiota of the piglets in both UniFrac indices (Table 2). These differences were observed graphically in the Principal Coordinates Analysis (PCoA) when Gut Region, but not Treatment were taken into account (Figure 3). It can also be observed main clustering between small and large intestine samples.

When we studied the effect of Treatment within each gut region, significant differences appeared at the. caecum level with Unweighted UniFrac (Figure 3) and at the colon level with both Unweighted and Weighted UniFrac (Figure 3). Antibiotic samples grouped in a cluster separated from control and *Allium* extract samples. Therefore, our treatment affected mainly to large intestine regions (Table 3).

### 3.3. Effects of Treatment on Piglets’ Productive Parameters

**Figure 4:**
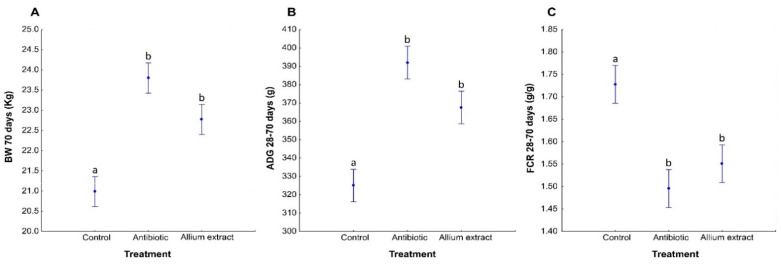
Average ± standard error of the mean of the Body Weight (BW) at 70 days of life (**A**), Average Daily Gain (ADG) (**B**), and Feed Conversion Ratio (FCR) (**C**) from 28 to 70 days of life of weaned piglets fed with control diet or antibiotic or *Allium* extract supplemented diets. Bars with different letter denote significant differences in treatment (LSD Posthoc test; *p* < 0.05).

Body weight significantly differed between treatments at day 70 (Table 4; Supplementary material: Table S2). Antibiotic and *Allium* extract fed piglets showed higher values of body weight than Control piglets (Table 4; Supplementary material: Table S2; Figure 4A). *Allium* extract group showed lower values of body weight than Antibiotic one, although this difference was marginally significant (LSD Posthoc test; *p* = 0.080).

During pre-starter stage (from 28 to 42 days), Antibiotic piglets had significantly more ADG and showed a better FCR than Control piglets, while *Allium* extract fed piglets showed intermediate values in both parameters. During starter stage (from days 43 to 70) Antibiotic and *Allium* extract showed higher values of ADG than Control piglets (Supplementary material: Table S2). Analyzing global stage (from 28 to 70 days), results showed that Antibiotic and *Allium* extract fed piglets significantly had higher ADG and lower FCR than Control piglets (Table 4; Supplementary material: Table S2; Figure 4B,C). No differences were observed between treatments in average daily feed intake (ADFI) or mortality (Table 4; Supplementary material: Table S2). 

## 4. Discussion

The addition of *Allium* extract in the diet of weaned piglets had a significant increase of body weight (BW) and average daily gain (ADG), and decrease of feed conversion ratio (FCR) respect to control diet. *Allium* extract fed piglets reached similar productive levels to those of antibiotic group (colistin + ZnO), but marginally significant differences appeared in BW and ADG. These beneficial productive changes were accompanied by significant changes in bacterial community as diminution of alpha diversity indices and significant changes in beta diversity in large intestine regions (caecum and colon). These changes in beta diversity only appeared in the caecum and colon but general behavior of gut microbiota was not affected by the treatment (no differences in interaction between Gut and Treatment; Table 2).

Alternatives to antibiotics that maintain productive parameters in pig breeding is essential to fight AMR spreading and improve animal welfare. Several alternatives to antibiotic growth promoters such as probiotics, prebiotics, enzymes, and plant extracts had been proposed to achieve it and also to reduce the probability of AMR appearing [2,21]. From this point of view, plant extracts or phytobiotic, which can modulate microbiota and increase productive parameters, appear to be good and safe alternative to antibiotics [46]. Different plant extracts improve animal performance, productive parameters, and induce changes in gut microbiome of animals. For instance, oregano oil in growing-finishing pigs improved growth performance and nutrient digestibility by modulating gut microbiota [47], and oregano oil had been also used together with carbohydrases in piglets, improving feed conversion ratio with respect to control and antibiotic growth promoter diets [23]. Other essential oils obtained from thyme and cinnamon improved body weight of weaning pigs and decreased the number of pathogens as *E. coli* in different gut regions [22]; and a mixture of essential oil from mint and cinnamon improved feed efficiency in piglets [24]. *Allium* extract, mainly garlic extract, had also been used in piglets’ diet in different studies, reducing diarrhea and inflammation caused by *E. coli* [27] and improving piglet performance and body weight [29]. In our study, piglet diet was supplemented with *Allium* extract, an extract of onion and garlic, of which the principal active components are propyl propane thiosulfinate (PTS) and propyl propane thiosulfonate (PTSO). Our results support the use of this phytobiotic compound in piglet diet given that animals showed a performance improvement characterized by an increase of body weight (BW) and average daily gain (ADG), and a decrease of feed conversion ratio (FCR) with respect to control group. Furthermore, in our study, piglets fed with *Allium* extract reached productive levels similar to those obtained using an antibiotic growth promoter (colistin) and ZnO. These results are promising as pork is one of the most consumed meat all over the world [9,48], thus *Allium* extract could be a good alternative to antibiotic growth promoters in pig diet given that improve productive parameters. Results obtained in other studies carried out with piglets suffering from diarrhea fed with plant extracts suggest that the growth promoting effects may be due to their antimicrobial activity [49,50]. This conclusion was also obtained in studies of [30] using both PTS and PTSO in swine, which had antimicrobial activity against different bacterial group in pig feces, especially against *Enterobacteriaceae* and other coliforms. Other studies pointed out that plant extracts increase productive parameters stimulating feed consumption [51], but other authors found that plant extracts decrease feed consumption [52]. Nevertheless, our results shown that piglets fed with *Allium* extract had similar levels of average daily feed intake (ADFI) compared to control and antibiotic groups.

Microbiome of intestine of pigs is dominated by Firmicutes, followed by Proteobacteria in the small intestine and Bacteroidetes in the large intestine [53,54,55,56]. At the class level, dominant classes of each phylum are Bacilli and Clostridia (Firmicutes), Bacteroidia (Bacteroidetes), and Gammaproteobacteria (Proteobacteria). Our results are consistent with these previous findings, especially at the phylum level. Other studies had shown that some Lactobacillus species play an important role in intestinal health of piglets by influencing intestinal physiology, regulating the immune system, and balancing the intestinal ecology of the host [57,58]. In our experiment, in caecum and colon, piglets supplemented with *Allium* extract showed similar levels of Bacilli versus control group, mainly due to the genus *Lactobacillus*. However, antibiotic group showed lower proportion of *Lactobacillus*, especially in the colon, showing that colistin and ZnO would have an effect on *Lactobacillus* depletion, whereas the genus *Prevotella* had an increase occupying its niche. This decrease in *Lactobacillus* abundance in colistin and ZnO piglets may be related to a depletion in carbohydrate levels in distal parts of the gut. In vitro studies have demonstrated that shifts in pig gut microbiome composition can be produced by changes in substrate structure [59]. Different *Allium* extracts produce changes in the physiology and histology of the gut of animals. In broilers, onion powder increased length, width, and surface area of intestinal villus [60]. In piglets, aged garlic extract improved body weight, the morphology of intestinal villi, and non-specific immune response [29]. Other studies using *Allium* extract in growing-finishing pigs showed an increase in productive parameters and an increase of short-chain fatty acids (SCFA) in feces, which is related to high *Lactobacillus* abundance in distal gut [61]. These changes may suggest that *Allium* extracts produce changes in the availability of some substrates necessary for the growth of beneficial bacteria. However, an in vitro study showed that PTSO extracted from *Allium* plants have antimicrobial activity against lactobacilli, bifidobacteria, *Bacteroides,* and Clostridia, and strongly reduce enterobacteria and coliforms in swine microbiota [30]. Whether PTSO and *Allium* extracts affect bacterial community directly or indirectly by change the substrate availability deserve future research.

Changes due to the supplementation of antimicrobials showed that main changes in bacterial community were produced in caecum and colon [62]. Our results are consistent with these previous findings, showing differences between treatments in large intestine regions (caecum and colon) in both alpha and beta diversity indices. These changes in bacterial community indices may be due to differential bioavailability of Garlicon in these intestinal regions. In vitro digestion studies of [63] showed that Garlicon bioavailability increases as it progresses in the gastrointestinal tract of pigs. Alpha diversity indices in the colon in the Antibiotic group were higher than in the *Allium* extract group. This reduction in alpha diversity levels could be related to the increase of body weight since reduction of alpha diversity has been associated with obesity in several human studies [64,65,66]. Different studies have found evidences that differences in microbial composition could be due to body weight [67] while other studies showed that changes induced by feed additives in gut microbiota can produce changes in body weight [68].

## 5. Conclusions

Our experiment supports the use of *Allium* extract supplemented in the diet of weaning piglets for successfully improving productive parameters such as body weight, average daily gain, or feed conversion ratio levels with respect to control diet. These beneficial effects in productivity correlates with significant changes in the bacterial community of the distal gut. These results are preliminary as further experiments are necessary to untangle whether *Allium* extracts directly affect the gut microbiota and hence the productivity parameters or whether the effects are directly on the bacterial community or on specific bacterial groups related to immune system or piglet’s health.

## Figures and Tables

**Table 1:** General Linear Models exploring the effects of treatment (control, antibiotic and *Allium* extract) and gut region in the different alpha diversity indices of the bacterial community of weaned piglets. D.f. refers to degree of freedom. The first number is the degree of freedom of the independent variable and the second one for the error term. Significant *p*-values (*p* < 0.05) are shown in bold.

Alpha Diversity Index	Control	*Allium* Extract	Antibiotic	Explanatory Variables	D.f.	F	*p*
Species richness	311.75 (27.38)	243.77 (18.71)	294.14 (41.60)	Treatment	2.61	4.03	**0.023**
				Gut Region	3.61	26.41	**<0.001**
				Gut Region × Treatment	6.61	1.57	0.171
Faith’s diversity index	24.53 (1.89)	20.14 (1.31)	22.59 (2.89)	Treatment	2.61	3.25	**0.046**
				Gut Region	3.61	22.11	**<0.001**
				Gut Region × Treatment	6.61	1.46	0.208

**Table 2:** General Linear Models exploring the effects of treatment, gut region, and their interaction in beta diversity indices of bacterial community of weaned piglets fed with control diet or supplemented with antibiotic or *Allium* extract. D.f. refers to degree of freedom. The first number is the degree of freedom of the independent variable and the second one for the error term. Significant *p*-values are shown in bold.

β-Diversity Distance Matrix	Explanatory Variables	D.f.	Pseudo-F	*p*
Unweighted UniFrac	Treatment	2.61	1.84	**0.001**
	Gut Region	3.61	7.88	**0.001**
	Gut Region × Treatment	6.61	1.06	0.303
Weighted UniFrac	Treatment	2.61	2.35	**0.044**
	Gut Region	3.61	9.14	**0.001**
	Gut Region × Treatment	6.61	1.02	0.412

**Table 3:** General Linear Models exploring the effects of treatment in beta diversity indices of bacterial community of weaned piglets fed with control diet or supplemented with antibiotic or *Allium* extract. D.f. refers to degree of freedom. The first number is the degree of freedom of the independent variable and the second one for the error term. Significant *p*-values are shown in bold.

	β-Diversity Distance Matrix	D.f.	Pseudo-F	*p*
Duodenum	Unweighted UniFrac	2.16	1.23	0.099
	Weighted UniFrac	2.16	0.25	0.977
Ileum	Unweighted UniFrac	2.15	0.93	0.502
	Weighted UniFrac	2.15	1.08	0.377
Caecum	Unweighted UniFrac	2.15	1.56	**0.007**
	Weighted UniFrac	2.15	1.48	0.191
Colon	Unweighted UniFrac	2.15	1.55	**0.017**
	Weighted UniFrac	2.15	4.18	**0.009**

**Table 4:** General Linear Models exploring the effects of treatment as factor, sex, and block as random factors and initial body weight as covariate, in weaned piglets fed with control diet or supplemented with antibiotic or *Allium* extract. BW refers to body weight, ADG to average daily gain, FCR to feed conversion rate, and ADFI to average daily feed intake. D.f. refers to degree of freedom. The first number is the degree of freedom of the independent variable and the second one for the error term. Significant *p*-values are shown in bold.

Dependent Variable	Control	*Allium* Extract	Antibiotic	Independent Variables	F	D.f.	*p*
Initial BW (28 days), kg	7.34 (0.35)	7.34 (0.33)	7.32 (0.37)	Treatment	<0.01	2.19	0.998
				Sex	0.21	1.19	0.653
				Room	0.93	1.19	0.346
BW 42 days, kg	10.50 (0.49)	10.87 (0.57)	11.40 (0.55)	Treatment	5.69	2.18	**0.012**
				Sex	4.98	1.18	**0.039**
				Room	29.67	1.18	**<0.001**
				Initial BW	116.14	1.18	**<0.001**
BW 70 days, kg	21.01 (0.76)	22.79 (0.98)	23.76 (0.92)	Treatment	14.59	2.18	**<0.001**
				Sex	8.96	1.18	**0.008**
				Room	2.46	1.18	0.134
				Initial BW	86.30	1.18	**<0.001**
ADG 28–70 days, g/d	325.25 (11.01)	367.71 (16.84)	391.53 (15.37)	Treatment	14.59	2.18	**<0.001**
				Sex	8.96	1.18	**0.008**
				Room	2.46	1.18	0.134
				Initial BW	26.13	1.18	**<0.001**
ADFI 28–70 days, g/d	562.73 (30.24)	566.26 (23.25)	583.79 (19.97)	Treatment	0.49	2.18	0.620
				Sex	2.14	1.18	0.161
				Room	18.45	1.18	**<0.001**
				Initial BW	11.22	1.18	**0.004**
FCR 28–70 days, g/g	1.73 (0.06)	1.55 (0.06)	1.50 (0.04)	Treatment	8.27	2.18	**0.003**
				Sex	1.64	1.18	0.216
				Room	11.88	1.18	**0.003**
				Initial BW	1.26	1.18	0.277
Mortality 28–70 days, %	5.00 (2.67)	2.50 (1.64)	1.25 (1.25)	Treatment	0.90	2.18	0.423
				Sex	0.08	1.18	0.787
				Room	1.52	1.18	0.233
				Initial BW	0.66	1.18	0.428

## Data Availability

Sequences are available in the Genbank-NCBI Sequence Read Archive (SRA) (https://www.ncbi.nlm.nih.gov/sra/), BioProject: PRJNA664026, Accession Nos. SAMN16192455 to SAMN16192544.

## Supplementary Materials

The following are available online at https://www.mdpi.com/2079-6382/10/3/269/s1: Table S1. Calculated composition and analysis (% per Kg of feed) of the diet used for piglets; Table S2. Average ± standard error of the mean of the Body Weight (BW) at 28, 42 and 70 days of life; and Average Daily Gain (ADG), Average Daily Feed Intake (ADFI), Feed Conversion Ratio (FCR) and mortality in different experimental stages and global stage of weaned piglets fed with control diet or *Allium* extract or antibiotic supplemented diets. Rows with different letter denote significant differences in treatment (LSD Posthoc test; *p* < 0.05); Figure S1. Microbial composition at genus level of piglets gut microbiota grouped by gut region and treatment. Genera in the legend are sorted from most abundant to lowest abundant.
