# Supplementary material for: Allium Extract Implements Weaned Piglet’s Productive Parameters by Modulating Distal Gut Microbiota
Source: Antibiotics (Basel). 2021 Mar 8;10(3):269. doi: 10.3390/antibiotics10030269 (PMC8001633; doi:10.3390/antibiotics10030269)
Supplement: Supplementary file 1 [file antibiotics-10-00269-s001.pdf]

Table S1. Calculated composition and analysis (% per Kg of feed) of the diet used for piglets.

Ingredients	Prestarter (28-42 d)	Starter (43-70 d)
Barley	15.00	20.00
Corn	0.00	13.39
Cornflakes	10.00	0.00
Wheat	34.24	35.00
Fullfat soybean	7.00	5.00
Soybean meal 47%	9.82	15.51
HP-300 (Soy extract)	8.00	4.00
Acid serum	10.00	0.00
Fat	1.92	2.57
Calcium carbonate	0.86	1.04
Monocalcium phosphate	0.89	1.20
Salt	0.34	0.52
Methionine	0.28	0.24
L-Lysine 50	0.90	0.84
L-Threonine	0.28	0.23
L-Tryptophan	0.06	0.06
Vit & Min Premix	0.40	0.40
**Calculated analysis, %**		
Ashes	5,57	5,12
Crude protein	19,00	18,50
Ethereal extract	4,92	5,32
Crude fiber	2,93	3,24
Neutral detergent fiber	9,51	10,64
Starch	34,96	40,23
Calcium	0,75	0,72
Total phosphorus	0,63	0,63
Available phosphorus	0,40	0,38
Sodium	0,24	0,22
Net energy, kcal / kg	2.500	2.470
Total lysine	1,39	1,31
Total methionine	0,52	0,48
Total Met+Cys	0,83	0,79
Total threonine	0,95	0,87
Total tryptophan	0,29	0,28
Digestible lysine	1,28	1,20
Digestible methionine	0,49	0,45
Digestible Met+Cys	0,76	0,72
Digestible threonine	0,86	0,78
Digestible tryptophan	0,26	0,25

Table S2. Average ± standard error of the mean of the Body Weight (BW) at 28, 42 and 70 days of life; and Average Daily Gain (ADG), Average Daily Feed Intake (ADFI), Feed Conversion Ratio (FCR) and mortality in different experimental stages and global stage of weaned piglets fed with control diet or *Allium* extract or antibiotic supplemented diets. Rows with different letter denote significant differences in treatment (LSD Posthoc test; $P < 0.05$).

Dependent variable	Control	*Allium* extract	Antibiotic
Initial BW (28 days), kg	7.34 (0.35)^{a}	7.34 (0.33)^{a}	7.32 (0.37)^{a}
BW 42 days, kg	10.50 (0.49)^{a}	10.87 (0.57)^{ab}	11.40 (0.55)^{b}
BW 70 days, kg	21.01 (0.76)^{a}	22.79 (0.98)^{b}	23.76 (0.92)^{b}
ADG 28-42 days, g/d	225.21 (16.71)^{a}	251.93 (29.99)^{ab}	291.25 (20.93)^{b}
ADG 42-70 days. g/d	375.27 (11.27)^{a}	425.60 (23.67)^{b}	441.67 (15.87)^{b}
ADG 28-70 days, g/d	325.25 (11.01)^{a}	367.71 (16.84)^{b}	391.53 (15.37)^{b}
ADFI 28-42 days, g/d	299.02 (29.28)^{a}	248.17 (14.10)^{a}	286.10 (16.40)^{a}
ADFI 42-70 days, g/d	694.59 (38.32)^{a}	725.31 (31.73)^{a}	732.64 (23.50)^{a}
ADFI 28-70 days, g/d	562.73 (30.24)^{a}	566.26 (23.25)^{a}	583.79 (19.97)^{a}
FCR 28-42 days, g/g	1.33 (0.09)^{a}	1.07 (0.12)^{ab}	0.99 (0.04)^{b}
FCR 42-70 days, g/g	1.85 (0.09)^{a}	1.74 (0.12)^{a}	1.67 (0.06)^{a}
FCR 28-70 days, g/g	1.73 (0.06)^{a}	1.55 (0.06)^{b}	1.50 (0.04)^{b}
Mortality 28-42 days, %	1.25 (1.25)^{a}	1.25 (1.25)^{a}	0.00 (0.00)^{a}
Mortality 42-70 days, %	3.88 (1.90)^{a}	1.25 (1.25)^{a}	1.25 (1.25)^{a}
Mortality 28-70 days, %	5.00 (2.67)^{a}	2.50 (1.64)^{a}	1.25 (1.25)^{a}

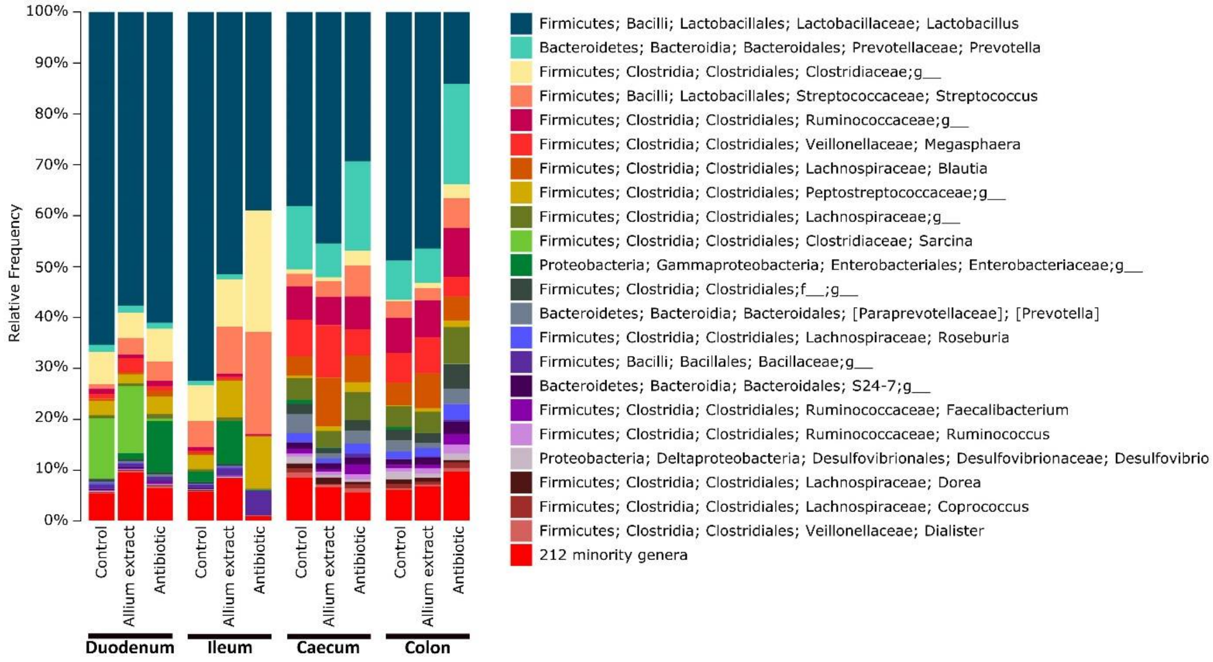

Figure S1. Microbial composition at genus level of piglets gut microbiota grouped by gut region and treatment. Genera in the legend are sorted from most abundant to lowest abundant.
